# Author response to: Factors influencing resilience to postoperative delirium in adults undergoing elective orthopaedic surgery

**DOI:** 10.1093/bjs/znac407

**Published:** 2022-11-25

**Authors:** Emma L Cunningham, Emily M L Bowman

**Affiliations:** Centre for Public Health, Queen’s University Belfast, Institute of Clinical Sciences, Belfast, UK; Centre for Public Health, Queen’s University Belfast, Institute of Clinical Sciences, Belfast, UK

*Dear Editor*

We are grateful to Mr Tokidis, Ms Mallappa, and Ms Altaf, on behalf of the Equality, Diversity and Inclusion Steering Group of the Association of Coloproctology of Great Britain and Ireland, for their interest in our paper^1^, and the pertinent point they raise.

We agree with and acknowledge the limitations of the National Adult Reading Test (NART)—namely that it was designed for people raised in Britain and speaking English as a first language.

At the time of study recruitment (2012–2014), our population in Northern Ireland was less heterogeneous than quoted by the correspondents, with 4.5 per cent of the population born outside the UK or Republic of Ireland, and 3.1 per cent speaking a main language other than English (https://www.nisra.gov.uk/publications/2011-census-key-statistics-northern-ireland). It is likely the population aged over 65 years from which we recruited was even more homogeneous. Although not documented, from memory, three participants did not speak English as their first language. To be eligible, participants had to be able to complete the assessments in English. We do not believe those ineligible or declining to participate were more diverse than those recruited.

Although the NART was appropriate for most participants in this study, we agree that future studies should be designed for, and include, more diverse populations.

Designing clinical research studies for, and with, people across the wealth of diversity in society increases the accuracy and relevance of findings. Assessments, in research and clinical spheres, must be reliable and valid across cultures, languages, and socioeconomic and educational spectra. We agree with the correspondents’ recommendation to use multiple assessment tools to gain as accurate results as possible. We are also working to better understand the effects of these spectra on cognitive tests.

## References

[1] Bowman EML , Cardwell C, McAuley DF, McGuinness B, Passmore AP, Beverland D et al Factors influencing resilience to postoperative delirium in adults undergoing elective orthopaedic surgery. Br J Surg 2022;109:908–911 35707934 10.1093/bjs/znac197 PMC10364747

